# Functional Analysis of a Missense Mutation in the Serine Protease Inhibitor *SPINT2* Associated with Congenital Sodium Diarrhea

**DOI:** 10.1371/journal.pone.0094267

**Published:** 2014-04-10

**Authors:** Nicolas Faller, Ivan Gautschi, Laurent Schild

**Affiliations:** Department of Pharmacology and Toxicology, University of Lausanne, Lausanne, Switzerland; Innsbruck Medical University, Austria

## Abstract

Membrane-bound serine proteases play important roles in different biological processes. Their regulation by endogenous inhibitors is poorly understood. A Y163C mutation in the *SPINT2* gene encoding the serine protease inhibitor Hepatocyte Growth Factor Inhibitor HAI-2 is associated with a congenital sodium diarrhea. The functional consequences of this mutation on HAI-2 activity and its physiological targets are unknown. We established a cellular assay in *Xenopus laevis* oocytes to study functional interactions between HAI-2 and candidate membrane-bound serine proteases expressed in the gastro-intestinal tract. We found that the wild-type form of HAI-2 is a potent inhibitor of nine gastro-intestinal serine proteases. The Y163C mutation in the second Kunitz domain of HAI-2 resulted in a complete loss of inhibitory activity on two intestinal proteases, prostasin and tmprss13. The effect of the mutation of the homologous Y68C in the first Kunitz domain of HAI-2 is consistent with a differential contribution of the two Kunitz domains of HAI-2 in the inhibition of serine proteases. By contrast to the Tyr to Cys, the Tyr to Ser substitution did not change the inhibitory potency of HAI-2, indicating that the thiol-group of the cysteine rather than the Tyr deletion is responsible for the HAI-2 loss of function. Our functional assay allowed us to identify membrane-bound serine proteases as cellular target for inhibition by HAI-2 wild type and mutants, and to better define the role of the Tyr in the second Kunitz domain in the inhibitory activity of HAI-2.

## Introduction

Membrane-bound or membrane-anchored serine proteases have lately emerged as a subfamily of 20 serine proteases that all share a conserved catalytic domain and a transmembrane domain [1]. They display diverse physiological and pathophysiological roles such as roles in skin and intestinal barrier integrity [2]–[5], processing of atrial natriuretic peptide [6], iron homeostasis [7]–[9], trophoblastic development [10], hearing [11], [12] and ion homeostasis [13], [14]. More elusive, however, are the identity and the roles of their physiological inhibitors.

The *SPINT1* and *SPINT2 genes* encode two Kunitz-type serine protease inhibitors called Hepatocyte Growth Factor Inhibitor HAI-1 and HAI-2. HAI-1, first purified from a stomach cancer cell line [15], is found as a complex with the membrane-bound serine protease matriptase in human milk [16]. Furthermore, genetic evidence supports an interaction between *SPINT1* and the *St14* gene encoding matriptase in mouse skin [17]. The membrane-bound HAI-2 and HAI-1, with their two inhibitory domains of Kunitz-type and their transmembrane domain, are highly homologous. However, HAI-2 lacks the LDL-receptor class A domain. HAI-2 was cloned from placental tissue, and from a gastric or pancreas cancer cell lines [18]–[20]. In cell-free *in vitro* systems, HAI-2 is a potent inhibitor of the membrane-bound serine proteases hepsin, prostasin, matriptase and tmprss13 [21]–[24].

The physiological role of *SPINT2* is incompletely characterized. In mice, *SPINT2* contributes to the appropriate development of the embryo as indicated by *SPINT2* knockout embryos showing clefting of the embryonic ectoderm, neural tube defects and defective placental branching morphogenesis; these defects can be rescued by the disruption of the *Prss8* and/or *St14* genes (encoding prostasin and matriptase respectively) [25]–[27]. In humans, various mutations in the *SPINT2* gene have been reported and shown to be linked to a syndromic form of congenital sodium diarrhea, indicating that *SPINT2* likely plays a role in intestinal ionic homeostasis [28], [29]. Among these mutations, a missense mutation substitutes a conserved tyrosine in the second Kunitz domain for a cysteine (HAI-2 Y163C). It has been shown that this mutation decreases the ability of HAI-2 to inhibit the prototype gastro-intestinal serine protease trypsin [28], [29]. The physiological partners of HAI-2 remain presently unknown.

Functional interactions between serine proteases and protease inhibitors are usually studied in cell-free *in vitro* systems. Here, we established a cellular assay using *Xenopus laevis* oocytes as a heterologous expression system to functionally assess the activity of candidate serine proteases and their inhibition by HAI-2 and its mutant Y163C. We found that HAI-2 was an efficient inhibitor of several membrane-bound serine proteases expressed in the GI tract. The *SPINT2* Y163C mutation associated with congenital sodium diarrhea induced a loss of inhibitory activity towards a limited number of serine proteases such as prostasin and tmprss13.

## Material and Methods

### Ethics Statement

Work done with animals was performed according to swiss national guidelines. Mice and *Xenopus laevis* were kept in an animal facility regulated by animal care rules of the University of Lausanne. All animals had access to food and water ad libitum. Protocols regarding sacrifice of the mice and surgical procedures on *Xenopus laevis* used in this study have been reviewed and approved by the Service de la Consommation et des Affaires Vétérinaires of the Canton of Vaud, Switzerland (authorization no. 2312 to LS).

### Quantitative PCR analysis

Tissues were removed from three C57BL/6 8 week-old mice. Duodenal, jejunal, ileal, caecal and colonic (proximal and distal) tissues were longitudinally opened and mucosal side was scraped with a razorblade to obtain a fraction enriched in mucosal cells. To avoid contamination by squamous epithelium, colonic tissues was scraped a few millimeters above the anus. Total RNA was extracted from mouse tissues using the RNeasy kit from Qiagen according to the manufacturer's protocol. 500 ng RNA (RNA from the 3 mice was pooled together) was reverse transcribed into cDNA using the PrimeScript RTreagent kit (TaKaRa). RNA was treated with DNAse I (Promega) to remove any possible traces of genomic DNA before the cDNA synthesis. qRT-PCR experiments were carried out on an ABI PRISM 7500 equipment (Applied Biosystems). PCR was performed in 96-well plates (Applied Biosystems) in 20-μl reactions that contained 10 μl of FastSYBR Green Master Mix (Applied Biosystems), 125 nM of each primer and 4 μl of cDNA (diluted 30 times). For each gene, standard curves were obtained using tissues with the highest abundance and 1/1, 1/5, 1/25, 1/625 and 1/3125 dilutions. The analysis of the slope of the standard curves showed a PCR efficiency between 1.70 and 2. These values were used for absolute quantification. Relative quantification using reference genes such as *Actin* or *Gapdh* was not used because detection levels of those genes varied up to a difference of 4 cycles between different mouse tissues. Each gene was assessed in duplicates in two independent experiments.

### Expression of human *Spint2* and human membrane-bound serine proteases in *Xenopus* oocytes

cDNA clones of human prostasin, tmprss4, matriptase, hepsin, tmprss2, tmprss11a, tmprss13, enteropeptidase and HAI-2 were obtained from the Mammalian Gene Collection (MGC). Human tmprss3 is a kind gift of Bernard Rossier (University of Lausanne). Membrane-bound serine proteases were selected based on their gastro-intestinal expression, as reported in the literature (see results). The other members of the membrane-bound serine proteases family were not tested because of a reported expression that appears to be restricted to specific tissues, even though we cannot rule out that they might display any gastro-intestinal expression. All cDNAs were subcloned into the pSD(BS)easy vector for expression in *Xenopus laevis* oocytes. A FLAG-tag sequence was added to HAI-2 using a single BstEII restriction site located in the sequence encoding the region between both Kunitz domains of HAI-2. PCR amplification was performed to add a tag of 8 histidines in the N-terminal part of tmprss13. Point mutations in HAI-2 cDNA were introduced by site–directed mutagenesis (Stratagene's QuickChange) to generate HAI-2 mutants Y68C, Y163C, Y68S, Y163S and double mutant Y68C/Y163C.

Stage V and VI healthy oocytes were isolated from ovarian tissue of *Xenopus laevis* and pressure–injected with 100 nl of cRNA solution. For the functional assay, oocytes were injected with α, β and γ subunits of rat ENaC cRNAs (0,11 ng of each subunit per oocyte) and with cRNAs of membrane-bound serine proteases and HAI-2 wild-type or mutants as indicated in the results section. In this heterologous expression system, we found that the effects of the serine proteases on ENaC as well as the effects of HAI-2 on the serine proteases were dose-dependent. To minimize artifacts due to overexpression, we determined for each serine protease the amount of cRNA to be injected for a robust proteolytic activation of ENaC similar to the activation by trypsin. We also determined the minimal amount of HAI-2 cRNA necessary to completely inhibit the effect of the protease. For the biochemical assay, oocytes were injected with cRNAs of His-tagged tmprss13 (1.7 ng) and FLAG-tagged HAI-2 (8.3 ng). This ratio tmprss13/HAI-2 is similar to the ratio used in the functional assay. Oocytes were kept at 19°C in a low Na^+^ (for the functional experiments with ENaC) modified Barth solution (MBS) containing (in mM): 10 NaCl, 0.82 MgSO_4_, 0.41 CaCl_2_, 0.33 Ca(NO_3_)_2_, 80 *N*-methyl-D-glucamine (NMDG), 2 KCl and 5 HEPES or a normal Na^+^ (for the biochemical experiments done without ENaC) modified Barth solution (MBS) containing (in mM): 85 NaCl, 1 KCl, 2.4 NaHCO_3_, 0.82 MgSO_4_, 0.41 CaCl_2_, 0.33 Ca(NO_3_)_2_, and 10 HEPES, 4.08 NaOH.

### Electrophysiology

Electrophysiological measurements were made 12 hours after injection except for experiments with tmprss3 and tmprss15, which were performed 30 hours after injection. ENaC-mediated Na^+^ currents were measured in oocytes using the standard two-electrodes voltage clamp technique using a Dagan TEV voltage clamp amplifier (Dagan, Minneapolis, MN), the Digidata 1322 digitizer, and the PClamp 9 data-acquisition and analysis package (Axon Instruments, Molecular Devices, Sunnyvale, CA). The two electrodes contained 1 M KCl solution. All electrophysiological measurements were performed at room temperature (22°C) in a perfusion solution containing (in mM) 120 NaCl, 2.5 KCl, 1.8 CaCl_2_-2H_2_O, and 10 HEPES-H^+^. The holding potential inside the oocytes was -100 mV.

### Data analysis

The epithelial sodium channel (ENaC) is highly sensitive to amiloride with an IC50 of 0.1 μM. ENaC activity was measured by the amiloride-sensitive Na^+^ current (I_Na_ *^+^*), defined as the difference between Na^+^ current obtained in the presence (10 μM) and in the absence of amiloride. ENaC activity is increased by a variety of serine proteases including trypsin [13], [30], [31], making ENaC an ideal tool for monitoring serine protease activity. We used trypsin (Sigma-Aldrich Chemie) 5 μg/ml in the perfusion solution for 2–3 minutes to define a maximal proteolytic stimulation on ENaC activity as measured by the increase in I_Na_ *^+^*. In each condition, sensitivity of ENaC to trypsin (defined as the relative trypsin-mediated increase in I_Na_ *^+^*) can vary depending on whether proteases with or without HAI-2 wt/mutants are co-expressed with ENaC and was quantified by dividing, for each oocyte, I_Na_ *^+^* after trypsin treatment by I_Na_ *^+^* before trypsin treatment. A relative trypsin-mediated increase in I_Na_ *^+^* close to one reflects resistance of ENaC to trypsin because of the presence of an activating co-expressed protease. When HAI-2 fully inhibits the co-expressed protease, relative trypsin-mediated increase in I_Na_ *^+^* has a value (>1) similar to control oocytes injected with ENaC alone.

### Statistical analysis

All data are represented as means ± SEM. Statistical significance was determined with one-way or two-way ANOVA followed by Dunnett or Tukey's multiple comparison tests and indicated in the legend of the figures.

## Results

### mRNA expression of *Spint2* and candidate membrane-bound serine protease along the mouse gastro-intestinal tract

We first identified potential targets for HAI-2 inhibition that are relevant for the pathogenesis of sodium diarrhea. We have limited our selection to members of the membrane-bound serine proteases family expressed in different gastro-intestinal (GI) tissues, because HAI-1, a highly conserved homolog of HAI-2 was reported as the physiological inhibitor of the membrane-bound serine protease matriptase. From the literature, nine membrane-bound serine proteases are expressed in GI tissues namely *Prss8* (encoding prostasin), *Tmprss4*, *St14* (encoding matriptase), *Hepsin*, *Tmprss2*, *Tmprss3*, *Tmprss11a*, *Tmprss13* and *Tmprss15* (encoding enteropeptidase) [11], [21], [32]–[44].

**Figure 1:**
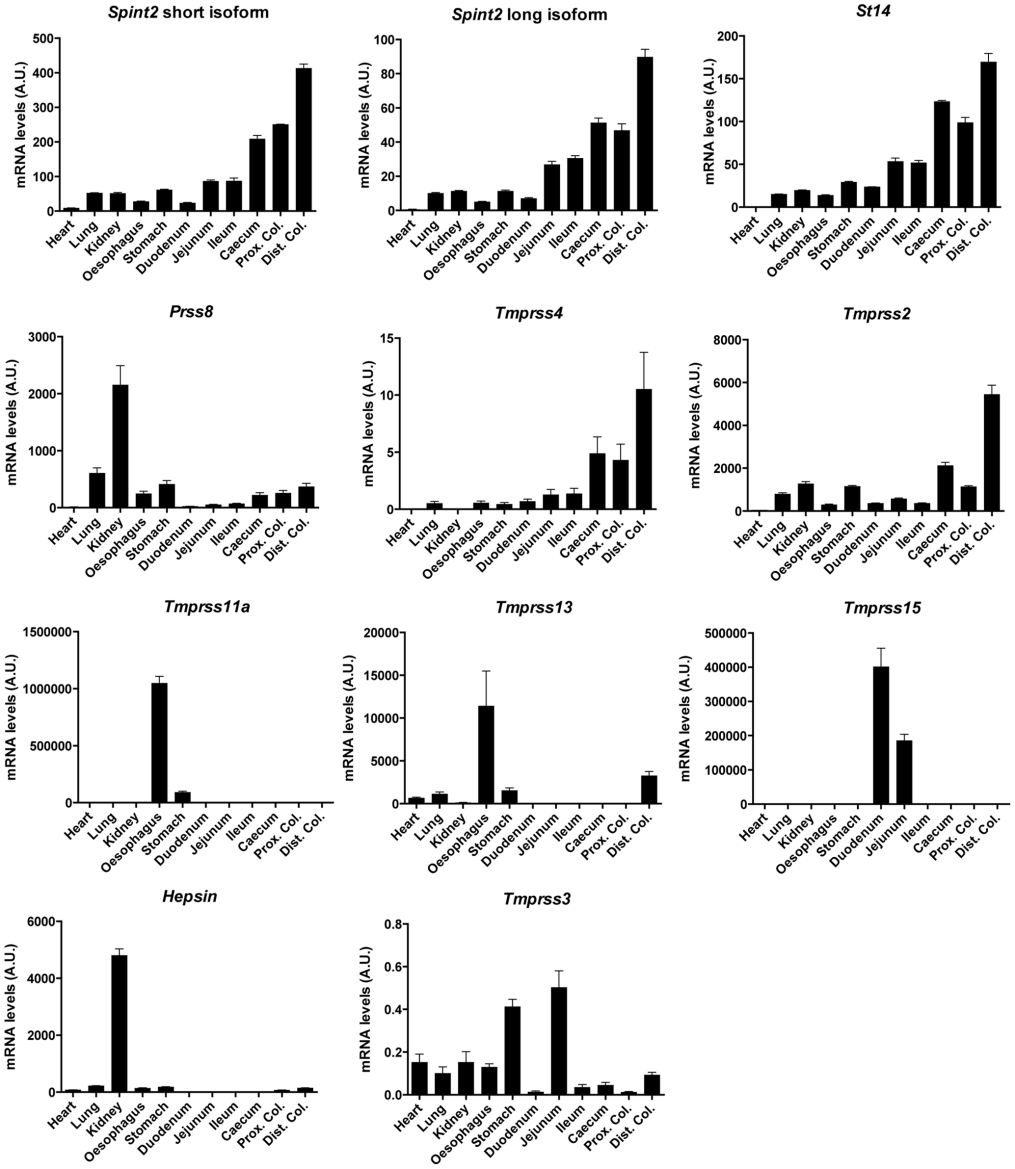
Tissue distribution of mRNA expression of *Spint2* and membrane-bound serine proteases. Quantitative RT-PCRs were performed on selected organs from three wild-type adult mice. From stomach to distal colon, tissues were scraped to get fractions enriched in mucosal cells. Each gene was assessed in duplicates in two independent experiments. Results are expressed as arbitrary units (A.U.) based on standard dilution curves (see Material and Methods).

The mRNA expression levels of *Spint2* and of the candidate proteases were determined in mouse GI tissues, and compared to heart, lung and kidney. Since *Spint2* is mainly expressed in epithelial cells [21], we took intestinal tissue fractions enriched in mucosal cells. Fig.1 shows that *Spint2* mRNA is found along the entire GI tract, but its expression increases in the distal part. As already reported, the short isoform *of Spint2* lacking the first Kunitz domain appears more abundant than the long (full-length) isoform in mouse (opposite findings are known in human) [45]. *St14* mRNA has an expression pattern similar to *Spint2*. *Prss8*, *Tmprss4* and *Tmprss2* are easily detected in the small and large intestines with a higher abundance in the distal part. The other candidates show a more restricted expression distribution along the GI tract: *Tmprss11a* is essentially expressed in oesophagus, *Tmprss13* in oesophagus and colon, *Tmprss15* in duodenum and jejunum. Although *Hepsin* is very abundant in the kidney, low expression levels are observed in the colon. Finally, *Tmprss3* mRNA is detected at very low levels in all tissues, with a higher expression in stomach and jejunum.

### Functional assay in *Xenopus laevis* oocytes

**Figure 2:**
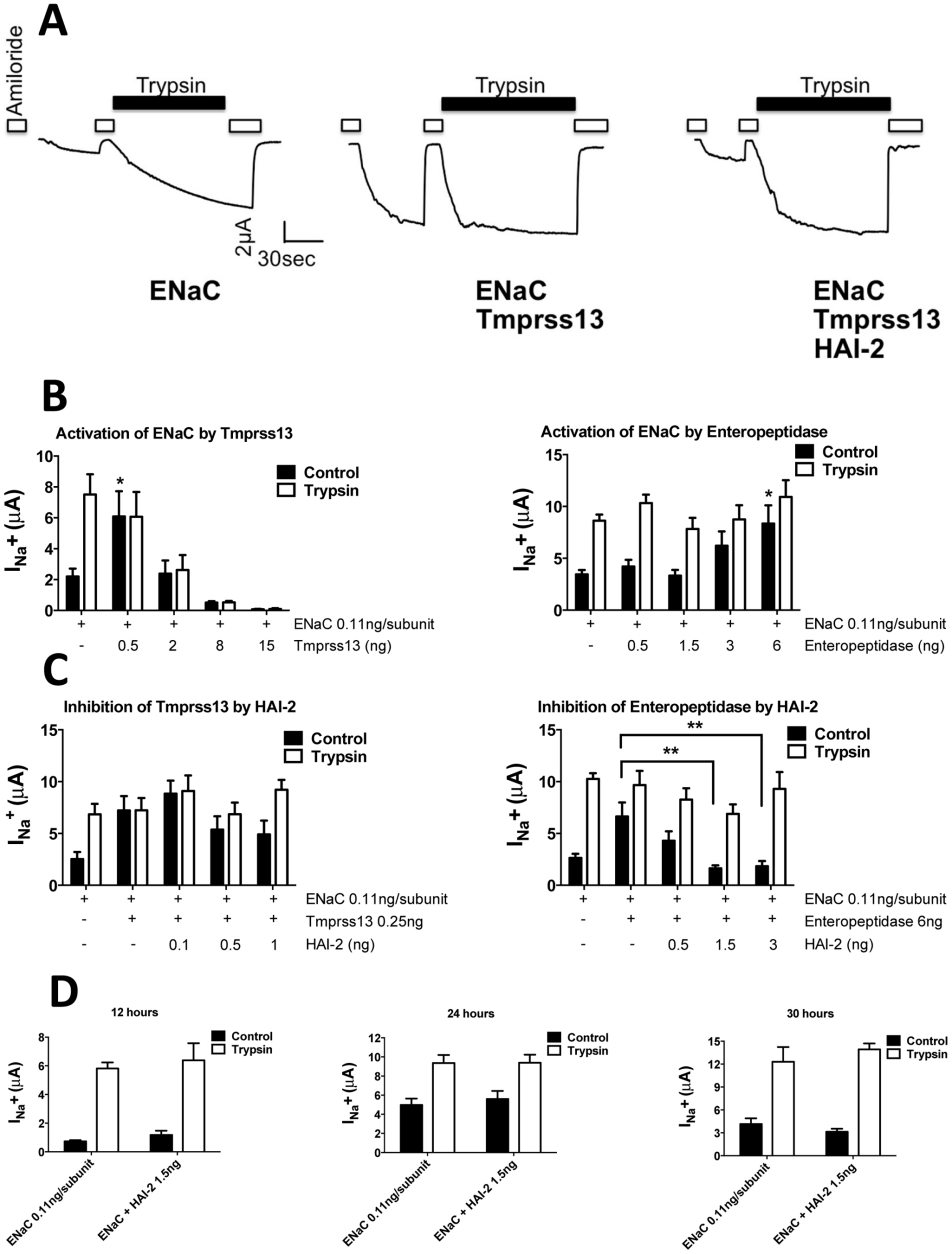
Validation of the functional assay using ENaC as a reporter gene. **A**, Representative recordings of amiloride-sensitive current (I_Na_^+^) in the presence (filled bars) or absence of trypsin (5 μg/ml), in *Xenopus* oocytes injected with 0.11 ng/subunit ENaC alone (left panel), with ENaC and 0.25 ng tmprss13 (middle panel) and with ENaC, tmprss13 and 1.5 ng spint2 cRNA (right panel). 10 μM amiloride was used to block the ENaC-mediated current. **B**, Effects of increasing the amounts of injected tmprss13 and enteropeptidase cRNAs on I_Na_^+^. I_Na_^+^ was measured in oocytes injected with ENaC with/without of tmprss13 or enteropeptidase as indicated. I_Na_^+^ was measured without (black bars) or with trypsin (5 μg/ml) perfused extracellularly (white bars) as a positive control for ENaC activation. **C**, Effects of increasing the amounts of injected spint2 cRNA to prevent the tmprss13- or enteropeptidase-mediated increase in I_Na_^+^ (left and right panels, respectively). **D**, Effect of spint2 on I_Na_^+^. I_Na_^+^ was measured 12, 24 and 30 hours after injection (left, middle and right panels, respectively) in three independent experiments. n = 6-9 measured oocytes per condition from 2 different batches for each experiment. Data are means ± SEM; *, p<0.05/**, p<0.01 compared to ENaC alone or ENaC + protease (as indicated) after two-way repeated measure ANOVA followed by Dunnett's multiple comparisons test.

To test whether HAI-2 inhibits our selected intestinal serine proteases in a functional cellular assay, we used as a functional readout the epithelial sodium channel ENaC and its unique property to be stimulated by a wide variety of serine proteases [30]. This allowed us to quantitatively assess by an electrophysiological approach the proteolytic activity of a serine protease, as an increase in ENaC mediated Na current. *Xenopus laevis* oocytes were injected with cRNAs encoding ENaC and the above mentioned serine proteases with or without HAI-2. The activity of the serine protease monitored by the increase in epithelial sodium channel ENaC activity, was measured as an inward current sensitive to amiloride, a known blocker of ENaC [46], [47]. The ENaC-mediated current was systematically compared with the maximal current obtained in the presence of trypsin, a well-established proteolytic agonist of ENaC. A typical electrophysiological recording is shown in figure 2A and illustrates the effect of the intestinal serine protease tmprss13 on ENaC-mediated currents (I_Na_^+^) and its inhibition by HAI-2. In an oocyte injected with ENaC alone, removing the ENaC blocker amiloride induces a discrete inward current which dramatically increases in the presence of trypsin (left tracing); co-injection of ENaC with tmprss13 increases the amiloride-sensitive current I_Na_^+^ to the level of I_Na_^+^ recorded in the presence of trypsin (middle tracing); finally, co-injection of ENaC tmprss13 and HAI-2 completely abolished the effect of tmprss13 on I_Na_^+^ (right tracing).

**Figure 3:**
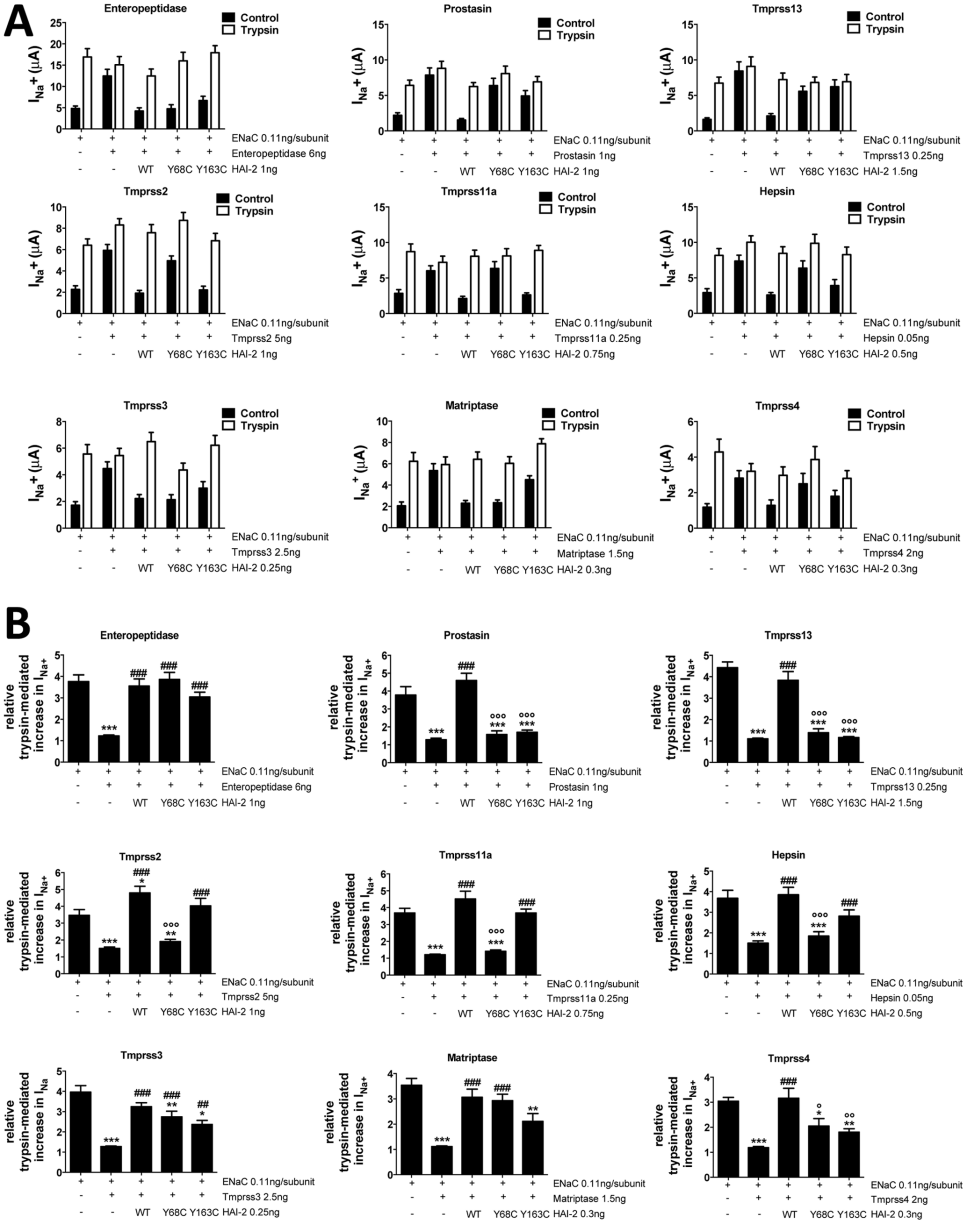
Functional analysis of interactions between HAI-2 (wt and mutants) and membrane-bound serine proteases. **A**, ENaC-mediated sodium currents (I_Na_^+^) were measured in *Xenopus* oocytes injected with ENaC with/without candidate serine protease and HAI-2 (wild-type or mutants Y68C and Y163C) as indicated. I_Na_^+^ was measured without (black bars) or with trypsin (5 μg/ml) perfused extracellularly (white bars) as a positive control for ENaC activation. n≥15 measured oocytes per condition from at least 2 different animals. Each protease was tested in at least two independent experiments. Data are means ± SEM. **B**, Relative trypsin-mediated increase in I_Na_^+^ was calculated by dividing, for each oocyte from experiments of panel A, I_Na_^+^ after treatment with trypsin by I_Na_^+^ before treatment with trypsin. Data are means ± SEM. */#/°, p<0.05, **/##/°°, p<0.01, ***/###/°°°, p<0.001, compared to ENaC alone, ENaC + protease or ENaC + protease + HAI-2 WT respectively after One-way ANOVA followed by Tukey's multiple comparisons test.

For some proteases such as tmprss13 we observed a dual effect on the activity of ENaC depending on the amounts of cRNA encoding the protease that were injected into the oocyte. Figure 2B compares the effects of increasing the amount of cRNA (i.e. the expression) of two proteases, tmprss13 (left panel) and enteropeptidase (right panel) on ENaC-mediated I_Na_^+^: co-injection of ENaC with 0.5 ng of tmprss13 cRNA increases I_Na_^+^ to the level obtained with trypsin but higher amounts of injected tmprss13 cRNA show a dose-dependent decrease in I_Na_^+^ in the presence and absence of trypsin. This observation is consistent with a progressive loss of ENaC activity at high levels of serine protease expression, likely due to an extensive proteolytic modification of the fully activated channel. For enteropeptidase a robust activation of ENaC activity to levels comparable with those obtained with trypsin could be obtained only with high doses of injected enteropeptidase cRNA up to 6 ng. We therefore systematically performed dose-response experiments for every serine protease tested to determine the minimal amount of the protease cRNA needed to stimulate ENaC-mediated I_Na_^+^ to levels comparable with those obtained for trypsin. All candidate serine proteases tested in our assay were functional as shown by the robust increase in ENaC-mediated I_Na_^+^ (see table 1 and figure 3). This effect varied from a 2 to 5 fold increase in ENaC activity depending on the protease and on the batch of oocytes used for the experiments.

**Table 1:** Increase in ENaC activity by membrane-bound serine proteases.

protease (ng of cRNA)	Fold increase in ENaC-mediated current
Tmprss2 (5 ng)	2.6±0.2 (trypsin: 3.5±0.3)
Tmprss11a (0.25 ng)	2.1±0.2 (trypsin: 3.7±0.3)
Enteropeptidase (6 ng)	2.6±0.3 (trypsin: 3.7±0.3)
Hepsin (0.05 ng)	2.5±0.3 (trypsin: 3.7±0.4)
Tmprss3 (2.5 ng)	2.6±0.3 (trypsin: 3.9±0.3)
Matriptase (1.5 ng)	2.6±0.3 (trypsin: 3.1±0.3)
Tmprss4 (2 ng)	2.4±0.4 (trypsin: 3±0.2)
Prostasin (1 ng)	3.6±0.4 (trypsin: 3.8±0.5)
Tmprss13 (0.25 ng)	5.2±0.8 (trypsin: 4.4±0.3)

Values were obtained from experiments in figure 3. Effect of trypsin is indicated in parentheses. Data are means ± SEM.

### Inhibition of serine proteases by HAI-2

We then evaluated the ability of HAI-2 to inhibit the different serine proteases shown in table 1. In preliminary experiments, as for the serine proteases, we determined the minimal amount of HAI-2 cRNA needed to completely reverse the effect of each protease tested. Typical experiments shown in figure 2C illustrate the inhibition of tmprss13 and enteropeptidase by HAI-2: 1 ng and 1.5 ng of HAI-2 cRNA are sufficient for a full inhibition of tmprss13 and enteropeptidase respectively as seen by a return of I_Na_^+^ to baseline and the recovery of the stimulatory effect of trypsin. We also verified that HAI-2 per se does not directly alter ENaC-mediated I_Na_^+^, neither the effect of trypsin on ENaC (fig.2D).

### Effect of the congenital sodium diarrhea-associated mutation of HAI-2

We analyzed the Y163C mutation in the 2^nd^ Kunitz domain of HAI-2 associated with congenital sodium diarrhea, and its effect on the inhibitory activity of the intestinal serine proteases. In addition, we have compared the Y163C mutation with its homologous Y68C mutation in the 1^st^ Kunitz domain to assess the relative roles of the two Kunitz domains in the proteolytic activity of HAI-2. Figure 3A compares the effects of the HAI-2 wt, HAI-2/Y68C and HAI-2/Y163C mutants on the activity of the serine proteases listed in table 1. For these experiments we used similar amounts of injected cRNAs assuming that the three HAI-2 constructs have comparable levels of expression in the oocytes. These experiments show that the wild type form of HAI-2 reverses the increase in the I_Na_ mediated by the serine proteases, indicating that HAI-2 efficiently inhibits all membrane-bound serine proteases tested. This suggests that HAI-2 is a functional inhibitor of various intestinal membrane-bound serine proteases.

The Y68C and Y163C mutations in the 1^st^ and 2^nd^ Kunitz domains have differential effects depending on the serine protease: the Y68C or Y163C mutations have no effects on the ability of HAI-2 to inhibit enteropeptidase, whereas they result in a loss of the inhibitory function of HAI-2 on tmprss13. On other serine proteases such as tmprss11a, only the Y68C but not the Y163C abolished the activity on HAI-2. A partial loss of function was observed with the Y163C mutation on the inhibition of matriptase by HAI-2. The data on figure 3A are expressed as absolute values of ENaC-mediated I_Na_^+^ reflecting serine protease activities. In order to compensate for variations in ENaC expression and protease activation of I_Na_^+^ among the different batches of oocytes, we assessed in figure 3B the relative efficiency of HAI-2 wild type and mutants on the different serine protease by quantifying for each oocyte the trypsin response on I_Na_^+^ (ratio I_Na_^+^ after trypsin treatment/I_Na_^+^ before trypsin treatment). When the relative trypsin-mediated increase in I_Na_^+^ is equal to one, there is no trypsin response indicating full effect of the serine protease; whereas a trypsin response ≥3 (value similar to that obtained for oocytes injected with ENaC alone) indicates a full inhibitory effect of HAI-2 on the serine protease. As mentioned before, both Y68C and Y163C mutants of HAI-2 are fully functional on enteropeptidase. On prostasin and tmprss13 both mutations Y68C and Y163C result in a near complete loss of HAI-2 function. On tmprss2, tmprss11a and hepsin, only the Y68C mutation leads to a loss of HAI-2 inhibitory activity. A partial loss of function with the HAI-2 Y163C mutant, and full activity with the Y68C mutant are observed on tmprss3 and matriptase, A partial loss of function is seen with both mutants on tmprss4. These experiments indicate that either the Y68C in the 1^st^ or the Y163C in the 2^nd^ Kunitz domain are sufficient to induce a loss of function of HAI-2 on prostasin and tmprss13 proteases. These latter proteases appear thus as interesting potential partners of HAI-2 the regulation of Na^+^ transport in the intestine. By contrast the Y68C mutation alone is sufficient to abolish the activity of tmprss11a, tmprss2 and hepsin, whereas the Y163C has no effect.

### Effect of the double mutant HAI-2 Y68C/Y163C, and of serine substitutions

**Figure 4:**
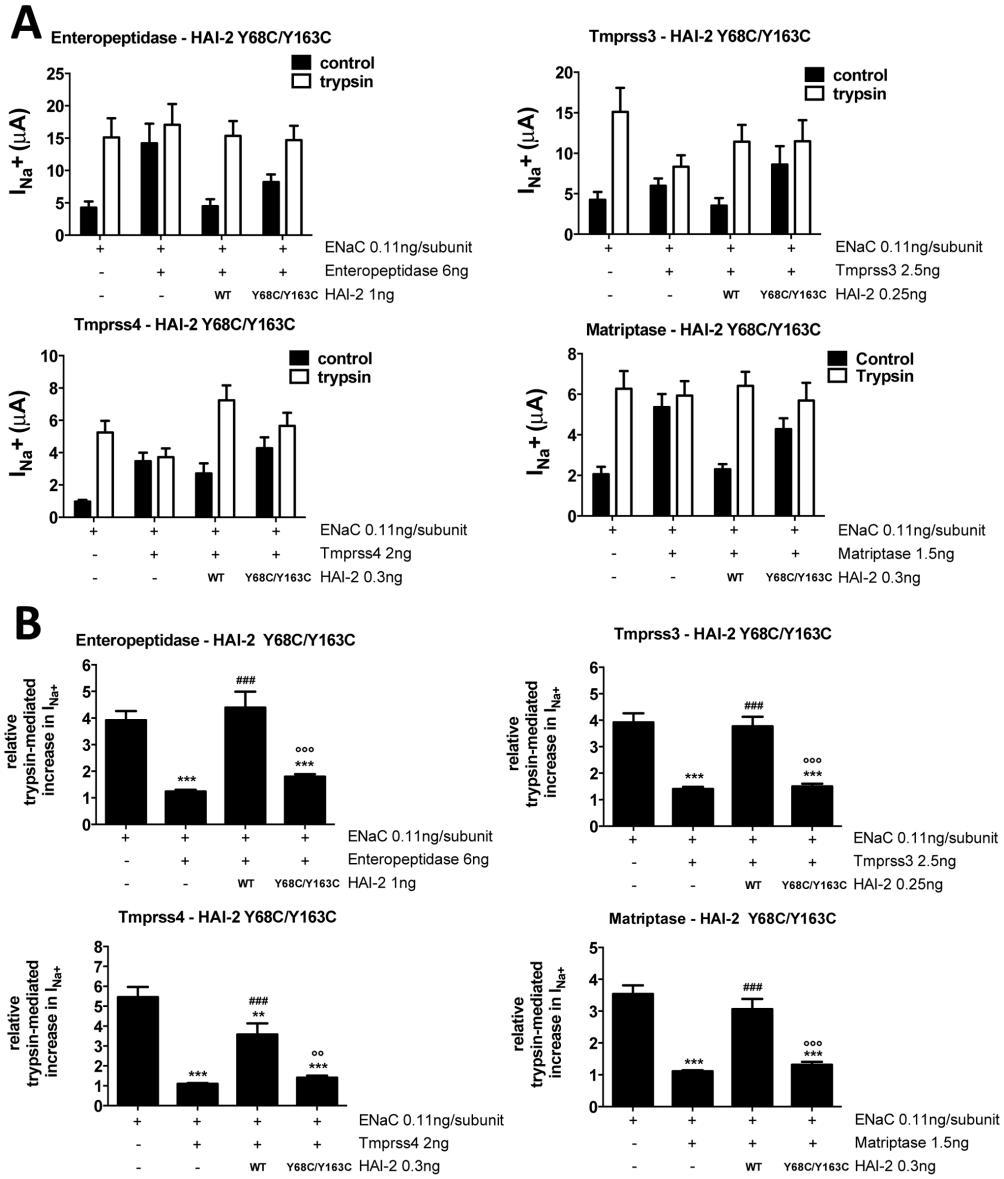
Effect of the double mutant HAI-2 Y68C/Y163C on enteropeptidase, Tmprss2, tmprss4 and matriptase. **A**, ENaC-mediated sodium currents (I_Na_^+^), were measured in *Xenopus* oocytes injected with ENaC with or without serine protease and HAI-2 (wild-type or double mutant Y68C/Y163C) as indicated. I_Na_^+^ was measured without (black bars) and with trypsin (5 μg/ml) perfused extracellularly (white bars) as a positive control for ENaC activation. n≥11 measured oocytes from 4 different animals. Each protease was tested in two independent experiments. Data are means ± SEM. **B**, Relative trypsin-mediated increase in I_Na_^+^ was calculated by dividing, for each oocyte from experiments of panel A, I_Na_^+^ after treatment with trypsin by I_Na_^+^ before treatment with trypsin. Data are means ± SEM. **/##/°°, p<0.01, ***/###/°°°, p<0.001, compared to ENaC alone, ENaC + protease or ENaC + protease + HAI-2 WT respectively after One-way ANOVA followed by Tukey's multiple comparisons test.

Four intestinal proteases, enteropeptidase, tmprss3, tmprss4 and matriptase are still partially, blocked by either HAI-2 Y68C or Y163C mutations. We assessed the effect of the double mutation HAI-2 Y68C/Y163C on these proteases. As shown in Fig.4, the Tyr to Cys mutations in both Kunitz domains nearly abolishes the inhibitory activity of HAI-2 on enteropeptidase, tmprss3, tmprss4 and matriptase, as shown on the measured ENaC-mediated I_Na_^+^ (fig.4A) or on the trypsin response (fig.4B).

**Figure 5:**
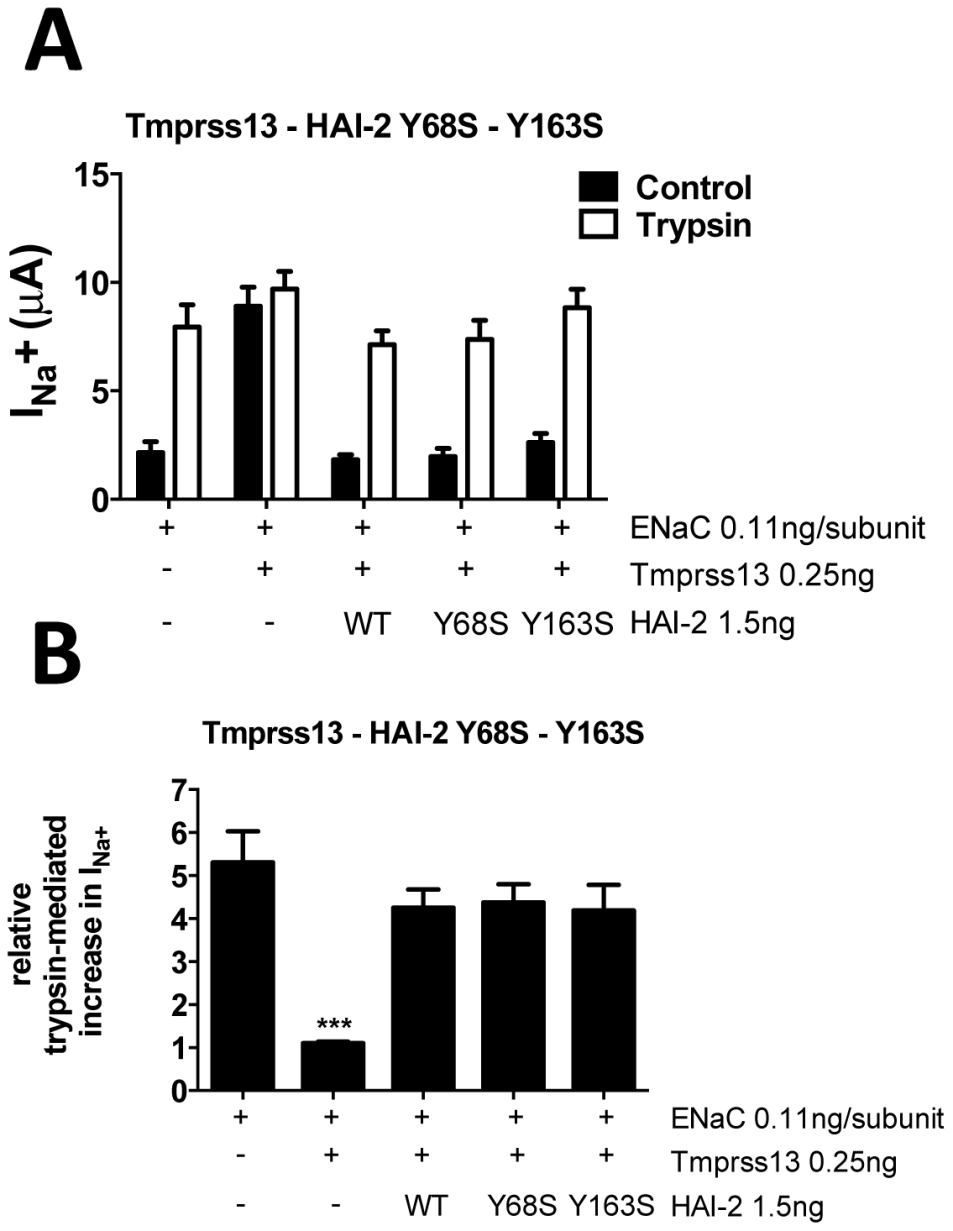
Effect of HAI-2 Y68S and Y163S mutations on tmprss13 activity. **A**, ENaC-mediated sodium currents (I_Na_^+^), were measured in *Xenopus* oocytes injected with ENaC, tmprss13 and HAI-2 (wild-type or mutant). I_Na_^+^ was measured without (black bars) and with trypsin (5 μg/ml) perfused extracellularly (white bars) as a positive control for ENaC activation. n≥14 measured oocytes from 4 different animals performed in two independent experiments. Data are means ± SEM. **B**, Relative trypsin-mediated increase in I_Na_^+^ was calculated by dividing, for each oocyte from experiments of panel A, I_Na_^+^ after treatment with trypsin by I_Na_^+^ before treatment with trpysin. Data are means ± SEM. ***, p<0.001, compared to ENaC alone, after One-way ANOVA followed by Tukey's multiple comparisons test.

To further understand the molecular basis of the Y68C and Y163C substitutions on the inhibitory capacity of HAI-2 towards a serine protease such as tmprss13, we asked whether this effect is due to the substitution of the tyrosine, which is highly conserved among HAI-2 orthologs from different species, or to the addition of a thiol group at position Y163. We mutated the Tyr68 and Tyr163 into a serine and assessed the effect of these mutations on the ability of HAI-2 to inhibit tmprss13. In contrast to the cysteine mutants, the serine mutants HAI-2 Y68S and HAI-2 Y163S retain their activity and are able to fully inhibit the activity of tmprss13 on ENaC (Fig.5). This suggests that the presence of the additional cysteine with its thiol side chain in the 1^st^ or 2^nd^ Kunitz domains is responsible for the loss of the inhibitory activity of HAI-2 on tmprss13.

## Discussion

In this study, we used a cellular assay to examine the functional interactions between HAI-2 wild type or mutants and different membrane-bound serine proteases expressed in the gastro-intestinal tract.

### An *in vitro* cellular assay to assess the activity of membrane-bound serine proteases

This assay uses the ENaC channel as the readout protein for the proteolytic activity of intestinal membrane-bound serine proteases. ENaC is a target for stimulation by a large number of membrane-bound serine-proteases. Prostasin, tmprss4, matriptase and tmprss3 have already been shown to activate ENaC [13], [34], [40]. Here, we report the activation of ENaC by hepsin, tmprss11a, tmprss13, enteropeptidase and tmprss2 [48]. An important aspect to consider in this assay is the dual effect of serine proteases on ENaC activity. The expected stimulatory effect on ENaC was observed at low levels of serine protease expression, as for the proteolytic cleavage of α and γ ENaC subunits at the cell surface. However, increasing the level of expression of serine proteases like tmprss13 resulted in the inhibition of ENaC activity (see Fig.2), suggesting an extensive proteolytic cleavage of the fully active ENaC that becomes incompatible with a channel function. This stresses the necessity in this assay to perform dose-response curves for every serine protease in order to quantitatively assess their effects on ENaC by both an increase in ENaC activity and a resistance to trypsin. The physiological relevance of the dual effects of these intestinal serine-proteases on ENaC activity still needs to be addressed in *in vivo* models.

### HAI-2 and membrane-bound serine proteases

We found that HAI-2 efficiently inhibits all intestinal proteases tested. The inhibition of hepsin, prostasin, matriptase and tmprss13 by HAI-2 has already been described [21]–[24] in cell-free assays. In addition, we also showed that HAI-2 inhibits tmprss4, tmprss2, tmprss3, tmprss11a and enteropeptidase.

**Figure 6:**
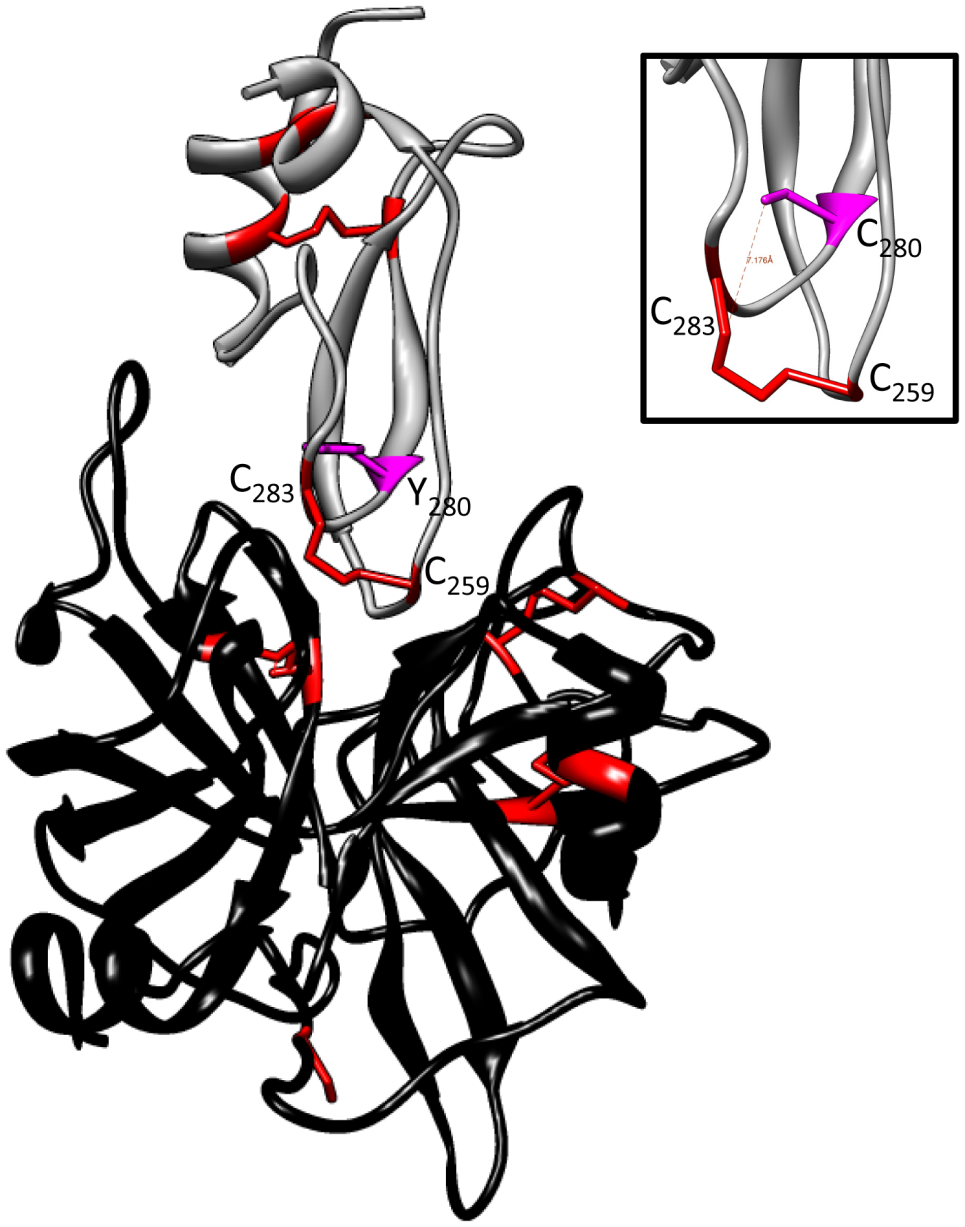
Structure of the catalytic domain of matriptase in complex with the 1^st^ Kunitz domain of HAI-1. The crystal structure of the complex was solved by Zhao et al. [49] and the atomic coordinates used for the figure were obtained from the Protein Data Bank (code 4ISO). The 1^st^ Kunitz domain of HAI-1 is shown in gray with the Tyr280 residue (magenta), and the cysteines (red) involved in disulfide bonds. The catalytic domain of the matriptase is represented in black with the cysteines (red) involved in disulfide bonds. *Insert*: substitution of the Tyr280 (magenta) in the KD1 of HAI-1with a cysteine pointing its side chain towards the Cys283.

The mutation of the conserved Tyr163 in the 2^nd^ Kunitz domain (KD2) of HAI-2, as well as the corresponding Tyr mutation in the 1^st^ Kunitz domain (KD1) alone or together resulted in a loss of function of HAI-2 against all the intestinal serine proteases tested in our assay. The crystal structure of the 1^st^ Kunitz domain of the HAI-1 (HAI-1KD1) in complex with the catalytic domain of matriptase provides useful information at the atomic level on the possible mechanism underlying the loss of function of the HAI-2 Y163 or Y68C mutant [49]. The KD1 of HAI-1 structure adopts a pear-shape structure formed essentially by 2 loops that are stabilized by three disulfide bonds (figure 6). This binding mode of HAI-1 KD1 is common for the inhibition of other serine protease of Kunitz type [50]. The Tyr in KD1 conserved in HAI-1 and HAI-2 is in a close vicinity of cysteines (C259 and C283 in HAI-1) involved in a disulfide bond at the interface between the HAI-1 and matriptase (figure 6). By contrast to the Cys 259 and 283 of HAI-1 that interact with the catalytic triad of matriptase, the Tyr is surrounded by hydrophobic residues and does not participate directly to the interface between HAI-1 and matriptase. Simulation of the Tyr substitution by either Cys or Ser in the HAI1-KD1 that reproduces the Y68C or the Y68S in our experiments, resulted in a drop of the stability of the residues surrounding the Tyr by 2.8 kcal/mol and 3.3 kcal/mol (calculated with FoldX3.0) [51]. Such a decrease in the stability of the loop at the interface of the HAI-1 and matriptase does not seem to affect the binding of KDI with matriptase since in our experiments the Y68S HAI-2 mutant retains its inhibitory activity. Our experiments indicate that the Tyr to Cys substitution has a more profound effect on the conformation of the HAI-1 loops at the interface with matriptase. One interpretation is that the thiol group of the substituted Y68C or Y163C may bridge with either cysteines C47/C71 or C142/C166 of the HAI-2 loop normally involved in a disulfide bond that fits the protease binding site. Such aberrant disulfide bond is expected to disrupt the conformation of the Kunitz domain loops of HAI-2 and its interaction with the protease; this hypothesis is supported by our results showing that the Y68C and Y163C of the HAI-2 almost completely suppressed the inhibitory activity of HAI-2 on tmprss13 and prostasin. Interactions between HAI-2 and membrane-bound serine proteases are probably complex and not limited to the interaction between one Kunitz domain and the catalytic domain of the serine protease as shown by the crystal. In our experiments we expressed full-length HAI-2 (with 2 Kunitz domains) and serine proteases that may have several accessory domains potentially involved in protein-protein interaction.

The analysis of the cysteine mutations in either or both Kunitz domains of HAI-2, support a differential contribution of the KD1 and KD2 in the inhibition of target serine proteases. The KD2 of HAI-2 is clearly less efficient than the KD1 for inhibition of tmprss11, tmprss2 and hepsin. The KD1 and KD2 are equally efficient in inhibiting tmprss13 and prostasin. For matriptase or tmprss4, mutations of both Kunitz domains are required for a complete inhibition by HAI-2.

Among the intestinal serine proteases tested, we observed a loss of function of HAI-2 activity against prostasin and tmprss13 by the Y163C mutation associated with the congenital sodium diarrhea mutant. These two proteases represent potential targets of HAI-2 in regulating intestinal Na^+^ transport. Prostasin shows significant mRNA levels in small intestine and colon like HAI-2, and recent genetic evidence shows that disruption of the *Prss8* (encoding prostasin) gene rescues the embryonic lethality of *Spint2* deficient mice [27]. Prostasin could also be a target of HAI-2 during embryonic development since children with the syndromic form of congenital sodium diarrhea also have dysmorphic features [28]. It should be mentioned that prostasin has been proposed to be an activator of ENaC in vivo [14] and notably in colon [52], [53]. Tmprss13, whose physiological role is unknown, is also an interesting candidate target for inhibition by HAI-2. Tmprss13 is significantly expressed at the mRNA level in distal colon. The proteases matriptase, tmprss3 or tmprss4 were only partially inhibited by the HAI-2 Y163C. We cannot exclude that these serine proteases represent physiological targets for HAI-2 and play a role in the pathogenesis of congenital sodium diarrhea. The *Spint2* expression pattern is for instance particularly similar to the *St14* gene that encodes matriptase (as shown in this study and in [21]). The functional targets of HAI-2 and the pathophysiological basis for the intestinal loss of Na^+^ ions in congenital sodium diarrhea still remain to be identified.

In summary, we developed a cellular assay in *Xenopus* oocytes to study functional interactions between membrane-bound serine proteases and inhibitors using ENaC as a reporter gene. *SPINT2*, whose mutations have been linked to congenital sodium diarrhea, appears to be a potent inhibitor. Being no longer blocked by the Y68C and Y163C HAI-2 mutants, prostasin and tmprss13 are interesting candidate partners of HAI-2 for maintaining Na^+^ homeostasis in the intestine. The functional effects of the cysteine or serine substitutions for the conserved Tyr in the KD1 and KD2 on the activity of HAI-2 are supported by the crystal structure of the complex formed by the HAI-1 homolog and matriptase.

## Supporting Information

Checklist S1 **ARRIVE Checklist.**
(DOC)
